# THE IMPACT OF PAID FAMILY LEAVE ON INFORMAL AND FORMAL CARE RECEIPT FOR MIDDLE-AGED AND OLDER ADULTS WITH DISABILITIES

**DOI:** 10.1093/geroni/igae098.3141

**Published:** 2024-12-31

**Authors:** Yuting Qian, Xi Chen

**Affiliations:** Yale University, New Haven, Connecticut, United States; Yale University, New Haven, Connecticut, United States

## Abstract

About one-sixth of middle-aged and older adults in the U.S. have difficulty with at least one activity of daily living (ADL), and this prevalence increases with age. Working caregivers face a trade-off between spending time earning income and providing care for their disabled family members. Recognizing this challenge, thirteen states and Washington, D.C. have implemented or recently passed mandatory paid family leave (PFL) programs, which provide partially paid leave for workers caring for ill family members. This paper analyzes the impact of PFL policies on both informal and formal care receipt among middle-aged and older adults with disabilities in the U.S., and the heterogeneous effects by family availability. We use data from the 1998-2018 public and restricted-use version of the Health and Retirement Study (HRS) and leverage the PFL programs implemented in California (2004), New Jersey (2009), and New York (2018) in a difference-in-differences (DiD) design. We find that, among individuals with disabilities who have both a spouse and children, PFL access is associated with an 8.0 percentage point increase in the likelihood that disabled individuals report receiving informal care from their children. We also show that PFL access significantly increases the receipt of formal care and increases the use of home care services and nursing home care for disabled individuals who have a spouse. Our findings demonstrate that PFL policies support healthy aging for middle-aged and older adults with disabilities, but its impact is limited for some vulnerable groups, such as disabled individuals with children only.

